# Cardiac Hemodynamics Abnormalities Induced by Intermittent Left Bundle-branch Block: A Case Report

**DOI:** 10.7759/cureus.2090

**Published:** 2018-01-20

**Authors:** Nestor O Neto, George C Fonseca, Gustavo G Torres, Luciano P Pinto, Fabio Mastrocola, William Santos R De Oliveira, Maria das Neves Barros

**Affiliations:** 1 Internal Medicine, Hospital Universitário Onofre Lopes/ufrn; 2 Cardiology, Hospital Universitário Onofre Lopes/ufrn; 3 Medical Student, UFRN; 4 Cardiology, Procape

**Keywords:** left bundle-branch block (lbbb), resynchronization therapy, dyssynchrony, heart failure, systolic time intervals, lbbb

## Abstract

The left bundle-branch block (LBBB) is related to worsening cardiac performance. We describe a case of a patient with an intermittent left bundle-branch block who underwent biventricular pacemaker implantation. We noted a detrimental effect on the performance of her left ventricle with the presence of the LBBB as compared to the performance after she underwent biventricular pacing or after reversal of LBBB (i.e., native rhythm with narrow QRS).

Her LBBB was accompanied by increased isovolumetric contraction time, longer pre-ejection period, and increased myocardial performance index (i.e., the Tei index) compared to her sinus rhythm with a narrow QRS complex with or under biventricular pacing.

## Introduction

Hemodynamic studies in heart failure patients with left bundle-branch block (LBBB) show that LBBB leads to worsening cardiac performance [1,2]. We describe the case of a patient with intermittent LBBB who received a biventricular pacemaker implantation, which allowed for the measurement of several echocardiographic parameters including biventricular pacing, sinus rhythm with a narrow QRS complex, and LBBB pattern. This case presents an opportunity to study the effects of LBBB associated with heart failure.

## Case presentation

A 55-year-old Caucasian woman presented to our hospital with a new onset dyspnea on exertion, lower extremity swelling, and orthopnea. Her electrocardiogram (ECG) showed sinus rhythm and a complete LBBB. She underwent a transthoracic echocardiogram (TTE) that revealed left ventricular (LV) enlargement and severe LV systolic dysfunction. She had no history of hypertension or alcohol abuse, and the results of her serology evaluation were negative for Chagas. A multislice computed tomography scan showed no lesions in the coronary arteries. She was diagnosed with idiopathic dilated cardiomyopathy.

Pharmaceutical treatment was initiated and optimized to reach the target doses of carvedilol, enalapril, spironolactone, digoxin, and furosemide. While the patient showed clinical improvement, she remained symptomatic after several months of optimized doses of these treatments. She subsequently experienced clinical worsening, remaining in New York Heart Association (NYHA) functional class III to IV. She was then admitted for implantation of a biventricular pacemaker.

During hospitalization, she showed rapid clinical improvement after reversal of the LBBB. An ECG showed sinus rhythm and a QRS complex interval of 110 ms. The medical team opted for not implanting the pacemaker at that time.

However, the patient returned 10 days later, presenting with clinical deterioration in NYHA functional class III. The ECG showed LBBB once more. She also brought the TTE performed on the day of discharge revealing LV enlargement with LV end-diastolic dimension of 67 mm, LV end-systolic dimension of 61 mm, LV end-systolic volume of 155 ml, an ejection fraction (EF) of 19% (by Simpson’s method), and Grade III diastolic dysfunction. We also noted a wide QRS complex in the ECG signal obtained during the TTE. Implantation of a biventricular pacemaker was scheduled.

**Figure 1:**
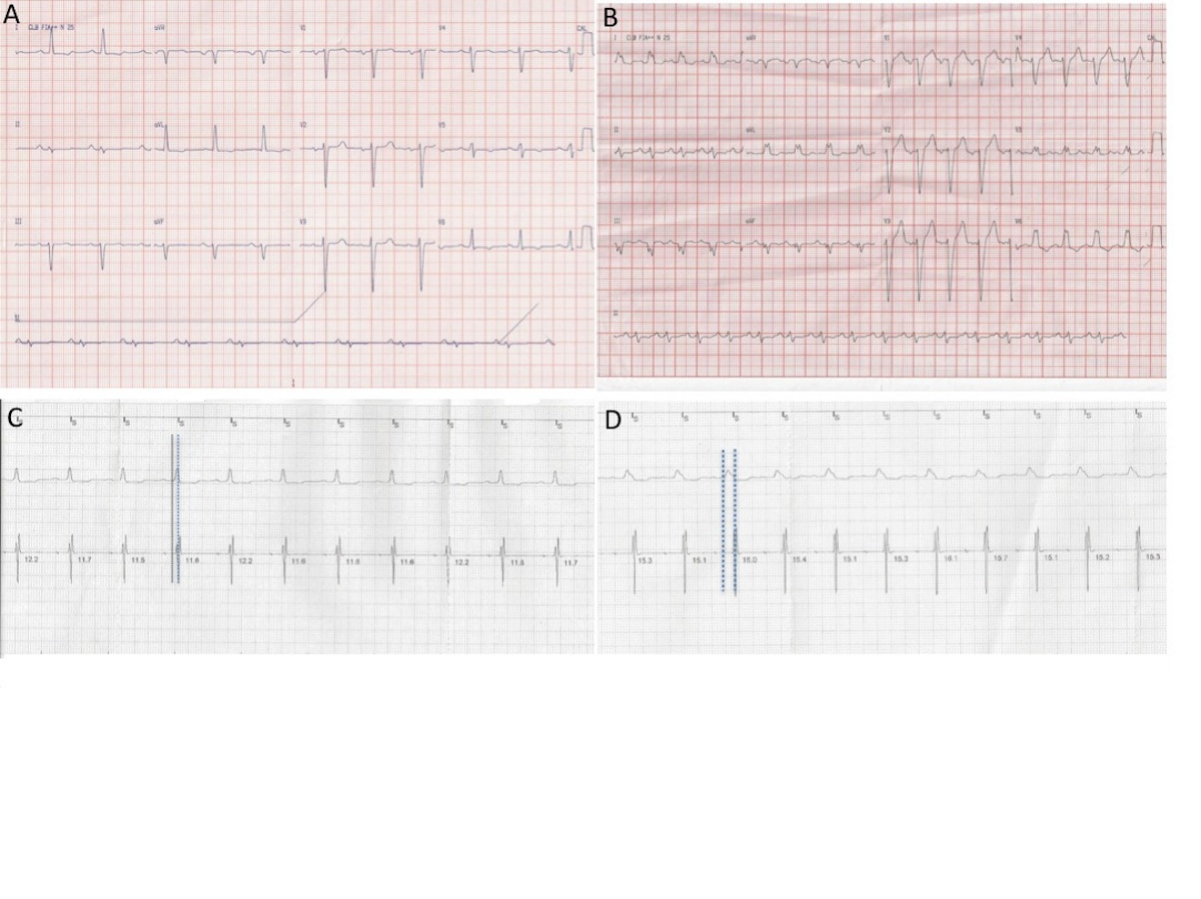
Electrical Parameters Twelve-lead ECG showing sinus rhythm and a narrow QRS (A) and sinus rhythm and left bundle branch with QRS of 160 ms (B). Intracavitary electrogram (EGM) obtained during implantation (electrode placed in the left posterolateral wall of the left ventricle) shows a Q-LV delay of 80 ms when the QRS is narrow (C). In the presence of left bundle-branch block, there is an increase of the delay of the EGM of the left ventricle with a Q-LV of 120 ms (D).

The implantation of the biventricular pacing was performed uneventfully, with the LV lead positioned through the coronary sinus in the posterolateral wall. During the procedure, we observed alternating narrow QRS complex intervals and LBBB. This allowed the electrode positioned in the posterolateral wall of the LV to capture the ECG of a narrow QRS sinus rhythm of 110 ms and LBBB (i.e., the QRS complex time was 160 ms). We documented an increase in the LV electrical delay. The interval time was defined as the time from the first deflection on a surface ECG to the local intrinsic activation at the LV stimulation site (Q-LV) after the installation of LBBB (Figure 1).

The patient showed clinical improvement after the pacemaker implantation, remaining in functional NYHA class II. We performed an echocardiogram to measure various parameters under three conditions: biventricular pacing (with a heart rate of 65 beats/minute), sinus rhythm with a narrow QRS complex, and LBBB pattern (Table 1).

**Table 1:** The echocardiographic parameters measured under three conditions: biventricular pacing, sinus rhythm with narrow QRS, and left bundle branch block. Abbreviations: CRT, cardiac resynchronization therapy; LBBB, left bundle-branch block; LVEF, left ventricular ejection fraction; PEP, pre-ejection period; ICT, isovolumetric contraction time; LVET, left ventricular ejection time; IRT, isovolumetric relaxation time.

Parameter	CRT	Narrow QRS	LBBB
LVEF (%)	19	20	20
PEP (ms)	170	150	210
ICT (ms)	89	90	143
LVET (ms)	280	300	280
IRT (ms)	143	140	150
Diastolic time (ms)	664	760	447
Myocardial performance index	0.83	0.77	1.05
Stroke volume (mL)	58	60	46
Cardiac output (mL/min)	3791	3608	3425

The sinus rhythm with a narrow QRS is the underlying rhythm, which we obtained after inhibiting the pacemaker. The LBBB pattern was obtained after atrial pacing at a rate of 75 beats/minute (the LBBB was rate-dependent).

**Figure 2:**
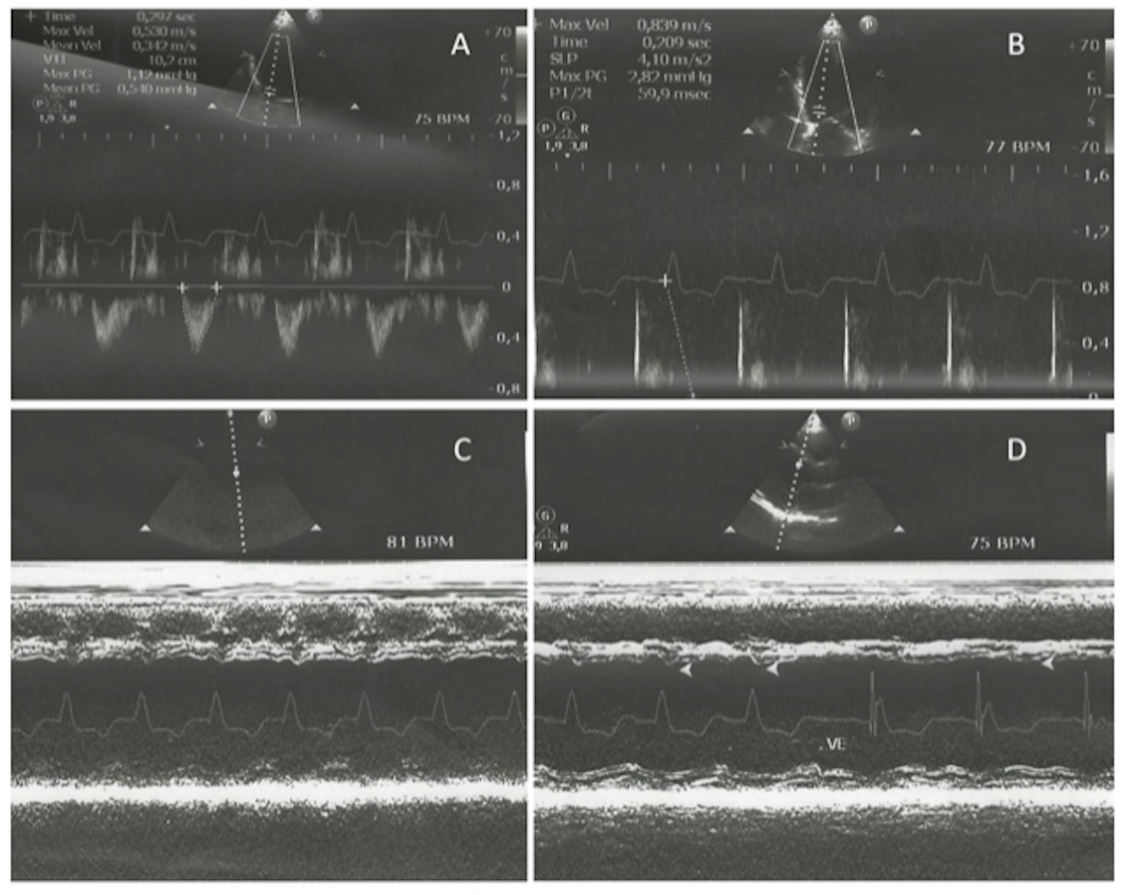
Echo Parameters Echocardiographic measures of velocity time integral (VTI) obtained in the LV outflow tract by pulsed Doppler (A) and of the pre-ejection period of the left ventricle (PEP) measured from the onset of the QRS complex to the beginning to the aortic ejection (B). M-mode tracings (C and D) showing abnormal septum motion (arrows) in the presence of left bundle-branch block, which disappeared after biventricular pacing.

The LVEF remained unchanged for each condition measured. There was a clear increase of the pre-ejection period (PEP), isovolumetric contraction time (ICT), and myocardial performance index in the presence of LBBB when compared to biventricular pacing and narrow QRS. We also observed abnormal interventricular septum motion during LBBB, which reversed at biventricular pacing or sinus rhythm with narrow QRS (Figure 2).

The patient presented good clinical function and echocardiographic response after the intervention. An echocardiogram done eight months later showed an increase in LVEF to 34% compared to the value of 19% before pacemaker implantation and an end-systolic volume reduction to 141 mL (before pacemaker implantation=155 ml). These parameters have been measured by Simpson’s method.

## Discussion

The induction of LBBB is accompanied by changes in LV systolic and diastolic intervals, with a delay of the onset and termination of systolic ejection and therefore, a reduction of the diastolic time, stroke volume, and arterial blood pressure [1-3].

In this case, we observed an increase of the LV electrical delay (posterolateral wall) and a detrimental effect at the LV during LBBB. These changes were reversed by biventricular stimulation or sinus rhythm with narrow QRS complex. LBBB increased the ICT, PEP, and myocardial performance index. LBBB increased the duration of systole and shortened the total time of diastole compared to the sinus rhythm with narrow QRS or biventricular pacing. Cardiac intervals and stroke volume (and cardiac output) presented similar values at sinus rhythm with narrow QRS and under biventricular pacing.

One study shows that the LV myocardial performance index, which is the ratio of the sum of the LV isovolumetric contraction time + LV isovolumetric relaxation time divided by the LV ejection time exhibited an acute and sustained improvement after cardiac resynchronization therapy (CRT) [4].

Bourassa et al. [3] reported findings similar to ours, with prolongation of ICT and shortening of systole after LBBB. In LBBB, the septum contracts early while the free wall is not yet activated; the activation of the free wall occurs when the septum is already relaxed leading to dyssynchronous LV contraction [5,6]. These effects lead to changes in the mentioned intervals that are corrected or mitigated by the CRT.

## Conclusions

The deleterious effects of LBBB on cardiac intervals are attenuated after their reversion to a narrow QRS rhythm or through biventricular pacing. The comparison of the parameters (i.e., ICT, PEP, and the LV myocardial performance index) measured in native rhythm and LBBB with a wide QRS complex and after implantation of biventricular pacing could be studied as a means of predicting the response to resynchronization therapy.
